# Increased burden of booster shots for COVID-19 amidst vaccine hesitancy in Pakistan

**DOI:** 10.1016/j.amsu.2022.104360

**Published:** 2022-08-22

**Authors:** Priyanka Mohan Lal, Omer Ahmed Shaikh, Laiba Imran Vohra, Aabiya Arif, Sidhant Ochani, Kaleem Ullah

**Affiliations:** a Department of Medicine, Ziauddin Medical University, Karachi, Pakistan; b Department of Medicine, Khairpur Medical College, Khairpur Mir's, Pakistan; c Department of Liver Transplantation, Pir Abdul Qadir Shah Institute of Medical Sciences, Pakistan

## Abstract

Pakistan is dealing with the fifth wave brought on by the new type, Omicron (or B.1.1.529) with 1,547,795 confirmed cases with 30,452 deaths of COVID-19, as reported by World Health Organization (WHO) from 2020 to 2022. Vaccination is the best tool to curb this pandemic and fight against the new variants as it reduces the likelihood that the disease will be severe. A two-dose regimen of the BNT162b2 vaccine provides 95% protection to people aged 16 years and above, against the novel coronavirus. However, like in other developing countries, the vaccination campaign in Pakistan is hampered due to vaccine hesitation. This might not be the last mutation the world shall face. As with any other virus, the corona-virus is also expected to mutate frequently in the future, making annual booster shots the only way to stay significantly immunized against this deadly virus. Communication and counseling are needed to build their trust while taking care of social inequalities in the population. The authorities must intensify their efforts and should address this very important issue.

Dear Editor,

In Pakistan, from January 3, 2020 to July 21, 2022, there have been 1,547,795 confirmed cases with 30,452 deaths of COVID-19, as reported by World Health Organization (WHO) [1]. Currently, Pakistan is dealing with the fifth wave brought on by the new type, Omicron (or B.1.1.529). This is the most recent coronavirus variety; discovered in South Africa and Botswana in November 2021. It has become Pakistan's dominant strain in recent days, particularly in Karachi, where the positive rate has surpassed 40%. Omicron has not spared a single city in Pakistan [2]. Health professionals issued a warning in July 2022, stating that the increase in new illnesses could result in the pandemic's sixth wave [3].

Due to world economic turmoil caused by travel bans and long-term lockdowns, the first-line defense mechanism against COVID-19 is not possible in the long term, especially in low-income countries like Pakistan, as the entry of new variants is inevitable. Vaccination is the best tool to curb this pandemic and fight against the new variants as it reduces the likelihood that the disease will be severe. A two-dose regimen of the BNT162b2 vaccine provides 95% protection to people aged 16 years and above, against the novel coronavirus [4]. However, like other developing countries, the vaccination campaign in Pakistan is hampered due to vaccine hesitation. From May 2021 to July 2022, Around 60% of the population of Pakistan has been immunized [5].

This vaccination campaign in the country is hampered because of vaccination hesitancy. Vaccine hesitancy has always been a notable phenomenon in the Pakistan population; a challenge faced till day during the polio vaccination. The term vaccine hesitancy refers to a delay in acceptance or refusal of vaccination despite the availability of vaccination services. The vaccine hesitancy is because of various local conspiracy theories. Since the early days of this pandemic, the misconceptions led by different conspiracy theories about COVID-19 and its vaccination have been on the rise. A few other factors also contribute much to this hesitancy i.e., low level of education, low socioeconomic status, lack of proper vaccine delivery system, lack of compliance, and access to the vaccine. All these factors make the general population unwilling to vaccination [6].

Due to spike protein mutation, the currently available vaccine has lost the eﬃcacy against the new N501Y variant. The mutation has made this variant more contagious than the previous variants. Almost 25% of the current cases in Pakistan were reported to be infected with the N501Y variant [7]. Because of the rapid mutating capability of this novel virus, the experts and researchers advise an annual booster shot for providing better protection against the new strain N501Y. A modified version of the vaccine is in the process, and soon will be available as a booster shot [8]. But, this will add even more burden on public health authorities in Pakistan too for convincing people for this additional annual booster dose.

However, this might not be the last mutation the world shall face. As with any other virus, the corona-virus is also expected to mutate frequently in the future, making annual booster shots the only way to stay significantly immunized against this deadly virus [9].

This pandemic has not only negatively impacted the economy and healthcare system, but it has also at its peak led to the cessation of polio vaccine campaigns, which subsequently led to a rise in reported polio cases [10].

It is of utmost importance to build the vaccination confidence of Pakistan's population, which mostly comprises rural areas and oppressed females. Hence, communication and counseling are needed to build their trust while taking care of social inequalities in the population. The authorities must intensify their efforts and should address this very important issue. Implementation of evidence-based communication, mass media strategies, and policy measures are required to combat this issue. Increasing misleading claims regarding the vaccine need to be neutralized by vigorous analysis of information by data or communications scientists, and publication of counter opinions from health professionals. Tailored and targeted strategies can be developed to address concerns of the general public; otherwise, the impact could span generations.

With vaccines and future annual booster shots being the only tool protecting Pakistan from a resurgence of COVID-19 cases, factors leading to vaccine hesitancy need to be addressed quickly to prevent Pakistan from having a never-ending battle against this pandemic. Therefore, vaccine hesitancy represents a massive obstacle to the successful control of the pandemic, unnecessarily perpetuating it and resulting in untold suffering and deaths.

## Ethical approval

None.

## Please state any sources of funding for your research

None.

## Author contribution

All authors contributed equally.

## Please state any conflicts of interest

None.

## Registration of research studies

N/A.

## Consent

None.

## Guarantor

All authors take responsibility for the work, access to data and decision to publish.
